# A Meta-Analysis of Reference Values of Leptin Concentration in Healthy Postmenopausal Women

**DOI:** 10.1371/journal.pone.0072734

**Published:** 2013-08-30

**Authors:** Xi Zhou, YanLan Chai, Ke Chen, YunYi Yang, Zi Liu

**Affiliations:** 1 Department of Radiation Oncology, The First Affiliated Hospital of the Medical College, Xi'an Jiaotong University, China; 2 Renmin Hospital, Hubei University of Medicine, Shiyan, Hubei, China; 3 Department of Physiology and Pathophysiology, Health Science Center, Xi'an Jiaotong University, China; University of Modena & Reggio Emilia, Italy

## Abstract

**Objective:**

There are numerous reports about the leptin concentration (LC) in postmenopausal women (PW). Changes in LC can elicit different clinical outcomes. We systematically analyzed the LC in PW.

**Methods:**

A search was conducted in original English-language studies published from 1994 to October 2012 in the following databases: Medline (78), Cochrane Center (123) Embase (505), Biological abstracts (108), Cochrane (53) and Science Finder Scholar (0). A meta-analysis was undertaken on the correction coefficient (*r*) between the serum LC and body mass index (BMI) for healthy PW across studies containing a dataset and sample size. Pre-analytical and analytical variations were examined. Pre-analytical variables included fasting status (FS) and sampling timing. Analytical variation comprised assay methodology, LC in those undertaking hormone replacement therapy (HRT) and those not having HRT as well as LC change according to age.

**Results:**

Twenty-seven studies met the inclusion criteria. Eighteen studies detected LC in the morning in a FS, 15 studies denoted the *r* between leptin and the BMI. A combined *r* was counted for the 15 studies (*r* = 0.51 [95% confidence interval (CI), 0.46–0.54], *P* = 0.025), and if sampling collection was in the FSat morning, a combined *r* was form 10 studies (*r* = 0.54 [95% CI, 0.45–0.54], *P* = 0.299) and heterogeneity was diminished. LC did not change between HRT users and non-users in 7 studies. Five studies analyzed changes in LC according to age.

**Conclusion:**

Based on all studies that investigated both LC and BMI, LC was positively correlated with the BMI. No studies established reference ranges according to the Clinical and Laboratory Standards Institute (CLSI) in healthy PW, and there was a wide variation in LC values. These differences suggest that caution should be used in the interpretation and comparison between studies.

## Introduction

The prevalence of obesity is the result of multiple factors. Obesity can lead to severe health problems and is a social and economic burden. Some genetic loci for obesity have been identified, including several energy homeostasis-related peptide hormones, such as leptin, cocaine-amphetamine-regulated transcript (CART) and ghrelin [1]. These hormones target special areas in the brain and regulate body metabolism; mutations in their loci or receptors can result in obesity [1]–[3]. Recently, disorders of the central nervous system were recognized as having a potential role in obesity [4]. Leptin, one of obesity relating factors, is an adipocyte-derived hormone important for fat metabolism, and leptin levels correlate with insulin resistance [5]. Leptin is also associated with reproductive functions [6], and immune responses [7], [8]. The central targets and mechanisms of leptin action have led to a detailed understanding after more than 10 years of research. Leptin crosses the blood–brain barrier (BBB) *via* saturable transport. Leptin has a role as a sensor of fat as part of a negative feedback loop that maintains a set point for fat stores within the body [9], [10]. The hormones act on specific centers in the brain that regulate the sensations of satiety, and these effects are more obvious than peripheral administration of leptin. Furthermore, leptin can improve depression-like behavior in animals by modulating synaptic plasticityin the hippocampus [11]. Based on the central action of leptin, it has been suggested that administration of leptin in the brain is more specific if it was used to treat obesity [4].

Numerous reports have focused on leptin expression in postmenopausal women (PW). Leptin expression has been associated with breast cancer [12], [13], hepatocellular carcinoma [14]–[16] and ovarian cancer [17], [18]. Hence, compressive analyses of leptin concentration (LC) is a basis to recognize these diseases in PW, specifically in breast cancer [12], [13] and ovarian cancer [17], [18] because its morbidity increases during menopause compared with before menopause [19].

## Methods

### Study design

This meta-analysis was conducted on literature published from January 1994 to October 2012. The databases searched were EMBASE, PubMed, Science Finder Scholar, Biological Abstracts and Cochrane. All studies were retrieved based on a search strategy in our meta-analysis using the following criteria: (i) study design – clinical cohort, cross-sectional and case–control studies were considered eligible; (ii) target population – healthy PW; (iii) specific definition of the methods used for the measurement of LC (plasma or serum), biochemical assay used [radioimmunoassay (RIA), enzyme-linked immunosorbent assay (ELISA)] and calculation of LC.

### Data extraction

Data were extracted from the articles using a specific data form. This form included information about search yield (key words: “normal” or “healthy”, “postmenopausal women” or “post menopause”, “serum” or “plasma”, “leptin concentration” or “leptin level” or “leptin value”). In total, 27 studies met the criteria for English language and healthy PW. None of studies gave the same sample size or LC range.

### Analyses

Intercooled Stata 12 for Windows was used for all data analyses. Meta-analyses of correlations were conducted using the method described by Hedges and colleagues [20], [21]. Briefly, using a Fisher's *r*→Z transformation to normalized 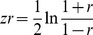, a combined correlation coefficient (*r*) was calculated for studies reporting multiple correlations between the subgroups studied. Then, transfer back was carried out using the transformation 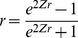, a variance of 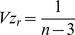 for the fixed-effect and 
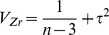
 for a random effect. A Q-statistic (Cochran's *Q*-test) was used to assess heterogeneity across studies, with significant heterogeneity noted as *P<*0.05. All of included studies were analyzed together and, if applicable, they were also analyzed by: (i) a combined *r* between leptin and the body mass index (BMI); (ii) participant with or without hormone replacement therapy (HRT) (Inclusion criteria for HRT were last menstrual period from 6 months to 1 year before the date of first visit (follicle stimulating hormone >35 IU/L); absence of any significant pathology; BMI<30 kg/m^2^; absence of contraindications to estrogen plus progestin therapy (EPT); no use of hormonal drugs in the past 6 months. Exclusion criteria for HRT were the presence of menstrual cycles or spotting; contraindications to EPT; hypersensitivity to progestogens and/or adhesive matrixes; obesity (BMI>30 kg/m2) or pathologic leanness (BMI <19 kg/m2); hypertension (borderline hypertension excluded); diabetes, glucose intolerance); (iii) LC change in accordance with PW age; (iv) RIA or ELISA method; (5) LC in pre-PW and PW. To determine reference ranges, the lowest concentration and highest concentration in the included studies were obtained. The weighted mean reference range was calculated for studies that used the same assay methodology. *P*<0.05 was considered significant.

## Results

### Summary of the included studies

**Figure 1:**
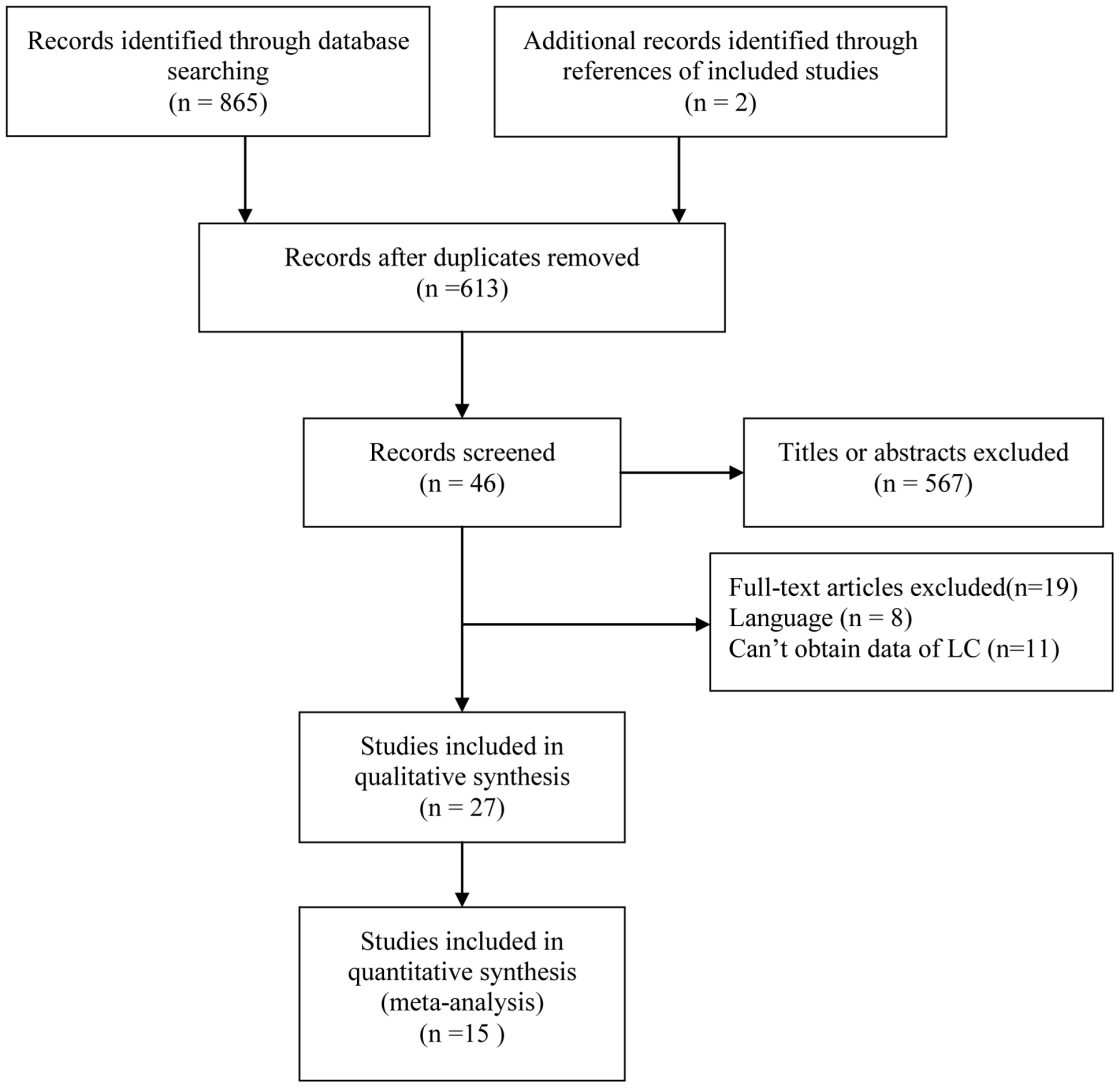
Flow chart from the identification of studies. **LC, leptin concentration.**

A detailed flowchart of the selection process is shown in Figure 1. The 27 studies that met the inclusion criteria are summarized in Table 1. The study design involved 20 cross-section studies [22]–[41], five fixed cohort studies [42]–[46] and two dynamic cohort studies [47], [48]. A total of 3,093 PW were in the included studies. Studies were characterized by total sample size, methods of LC detection, sampling conditions, and *r* with the BMI. In these studies, RIA (*n = *24) was the more commonly used assay methodology relative to ELISA (*n* = 3), there was no clear difference in LC between the two methodologies. Of the 27 studies, 18 studies detected LC in the morning in a fasting state (FS) (Table 1). Five studies gave the complete mean [SD] and range of LCs (1.01–80.8 ng/mL) (Table 2). Fifteen studies analyzed the *r* between leptin and the BMI of PW. Seven studies compared the LC between PW using HRT and those not using HRT. One study detected LC continually during a period of slimming in PW. None of the studies clearly explained the detection of free LC.

**Table 1:** Basic characteristics of all included studies and the *r* with the BMI.

Study	Number	Sampling status	Sampling timing	*r*	Method
Larsson *et al*. [22]	130	Fasting	Morning		ELISA
Haffner *et al*. [23]	28	Fasting	–	0.793	RIA
Goulding *et al*. [24]	54	Fasting	Morning		RIA
Kristensen *et al*. [42]	295	Fasting	Morning	0.475	RIA
Di Carlo *et al*. [47]	164	Fasting	Morning	0.53	RIA
Hadji *et al*. [25]	352	Fasting	Morning		RIA
Hadji *et al*. [26]	216	Fasting	Morning	0.47	RIA
Blain *et al*. [27]	167	Fasting	Morning	0.41	RIA
Cagnacci *et al*. [43]	148	Fasting	Morning	0.5745	RIA
Douchi *et al*. [28]	75	–	–		RIA
Hadji *et al*. [29]	94	Fasting	Morning		RIA
Lambrinoudaki *et al*. [30]	22	–	Morning	0.726	RIA
Munoz *et al*. [31]	80	Fasting	Morning		RIA
Sahin *et al.* [32]	100	–	–	0.356	ELISA
Shaarawy *et al.* [33]	90	Fasting	Morning		RIA
Ushiroyama *et al*. [34]	115	Fasting	Morning	0.514	RIA
Carlo *et al*. [44]	122	Fasting	Morning	0.562	RIA
Cordero-Maclntyre *et al*. [35]	39	–	–	0.4	RIA
Ayub *et al*. [36]	34	Fasting	–	0.2	RIA
Bednarek-Tupikowska *et al*. [48]	154	Fasting	Morning	0.52	RIA
Jaleel *et al*. [37]	80	Fasting	Morning		RIA
Rolland *et al*. [38]	76	Fasting	Morning		RIA
Carlo *et al*. [45]	120	Fasting	Morning	0.564	RIA
Meyers *et al*. [39]	114	Fasting	–		RIA
Khokhar *et al*. [40]	9	Fasting	–		ELISA
Wu *et al*. [41]	25	Fasting	Morning	0.765	RIA
Soni*et al*. [46]	200	Fasting	–		RIA

**Table 2:** Range of LCs of PW in the included studies.

Study	Lower concentration (ng/mL)	Upper concentration (ng/mL)
Goulding *et al*. [24]	1.0	80.8
Blain *et al*. [27]	2	66
Douchi*et al*. [28]	1.8	48.3
Munoz *et al*. [31]	5.2	63.6
Lambrinoudaki *et al*. [38]	2	54

Six studies specified the sampling population (2 studies in Caucasians [22], [43], 2 studies in Japanese subjects [28], [34], 1 study in Mexican–Americans and non-Hispanic whites [23], and 1 study in Caucasian and Afro-Americans [31]. One of the included studies noted different LCs in different races [31]. One report stated that racial differences were the reason for heterogeneity [49], a factor that must be borne in mind if combining LC values across studies.

### The BMI and LC of PW

**Figure 2:**
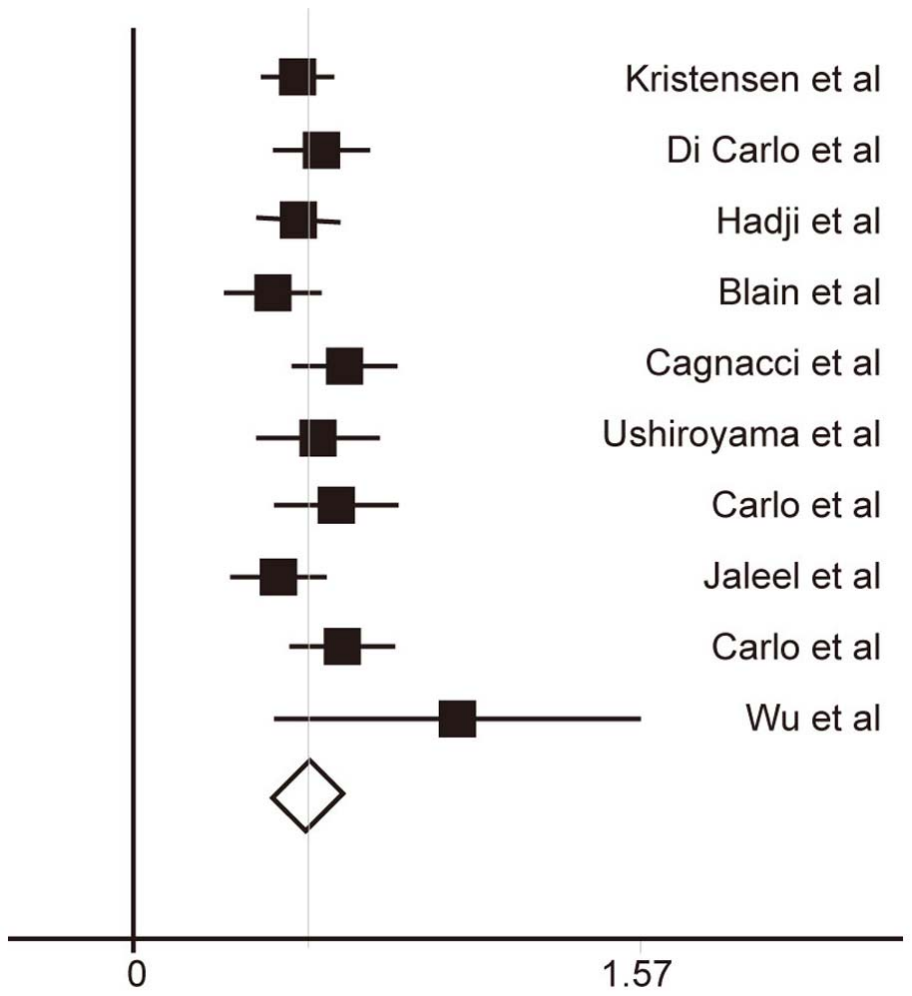
Forest plot indicating the *r* between LC and body mass index (BMI) of postmenopausal women (PW). Horizontal lines denote 95% confidence interval (CIs); the diamond indicates the combined r with the corresponding 95% CI.

Leptin is produced by adipose tissue. It is hypothesized that its level is higher in the obese than in the normal-weight population. In the 15 studies that gave the *r* between the BMI and LC, the *r* was from –0.075 to 0.793, and the combined *r* was 0.51 ([95% confidence interval (CI), 0.46–0.54], Q = 26.08, *P* = 0.025) in a fixed model and ([95% CI, 0.46–0.56], Q = 26.08, *P* = 0.025) in a random model. If we ignore the timing of sample collection in the 11 studies, then the combined *r* was 0.52 ([95% confidence interval (CI), 0.46–0.53], Q = 25.14, *P* = 0.027) in a fixed model and ([95% CI, 0.46–0.55], Q = 25.23, *P* = 0.026) in a random model, and after ignoring the sampling status of the 11 studies, the the combined *r* was 0.52 (95% CI, 0.46–0.52, Q = 25.34, *P* = 0.024) in a fixed model and (95% CI, 0.46–0.53, Q = 25.31, *P* = 0.026) in a random model. However, a combined *r* was taken from the 10 studies in which samples were collected in the FS in the morning [26], [27], [34], [41]–[45], [47], [48]; the combined *r* was 0.54 ([95% CI, 0.45–0.54], Q = 10.67, *P* = 0.299) in a fixed model and ([95% CI, 0.46–0.54], Q = 10.67, *P* = 0.299) in a random model, but heterogeneity diminished (Figure 2). LC explained 21% of the variation in the BMI.

### HRT and LC

HRT is recommended for use in PW. It can reduce some syndromes in PW [50]–[52]. Findings from the Women's Health Initiative (WHI) and other studies suggest that individual formulation for PW is more appropriate [53]–[55]. In 7 studies of the included studies (Table 3), there were no difference in LC in 5 studies and LC decreased in 2 studies after comparison of those using HRT and those not using HRT. However, in the study by DiCarlo *et al*. [47], the authors did not consider the effect of obsesity on LC, so their conclusion was not robust. In the study by Carlo *et al*. [45], LC did not change from the initiation of the study to that after 12 months of HRT. The authors also mentioned that the duration of HRT did not affect LC [31], [47], and concluded that HRT did not affect LC.

**Table 3:** LC in healthy PW and HRT users in the included studies.

Study	Duration of HRT	Comparison between PW using HRT and those not using HRT*
Haffner *et al*. [23]	7 years	N
Kristensen *et al*. [42]	5 years	N
Di Carlo *et al*. [47]	21.5–51 months	Lower in HRT group (p = 0.004)
Hadji*et al*. [25]	–	N
Lambrinoudaki *et al*. [30]	–	N
Munoz *et al*. [31]	1–72 months	N
Carlo *et al*. [45]	12 months	Lower in HRT group (p<0.05)

* N: no difference.

### LC and age

The age range of subjects in the included studies was ≈46–90.5 years. Five studies measured the *r* between age and LC; the age was ≈49–68 years (Table 4). Two studies found a positive correlation and two studies did not find a correlation between them. One study showed a negative correlation. Even if a meta-analysis cannot be done, the factor of age very weakly affects the LC.

**Table 4:** *r* between LC and age in PW in the included studies.

Study	Age [SD]	*r* *
Douchi *et al*. [28]	61.7 [7.7]	N
Sahin *et al*. [32]	55.1 [6.3]	N
Ushiroyama *et al*. [34]	53.3 [1.5]	0.25
Rolland *et al*. [38]	54.14 [4.24]	0.23
Khokhar *er al*. [40]	52.78 [4.92]	–0.081

* N: no correction.

Nine studies analyzed the LC in pre-PW and PW [23], [26], [28], [29], [36], [40], [41], [47], [48]. Three studies found no difference between them [23], [28], [48], 4 studies found that LC was higher in PW [26], [41], [43], [47] and the other 2 studies found LC was lower in PW [36], [40]. One of the 9 studies compared LC in pre-PW, peri-PW and PW [41]. The authors found that LC increased after the menopause, and that it did not differ between peri-PW and PW. A meta-analysis of subgroups could not be carried out because of appreciable heterogeneity. However, combination of the data about LC changing according to age, one could conclude that age did not affect LC very much.

### Publication bias

**Figure 3:**
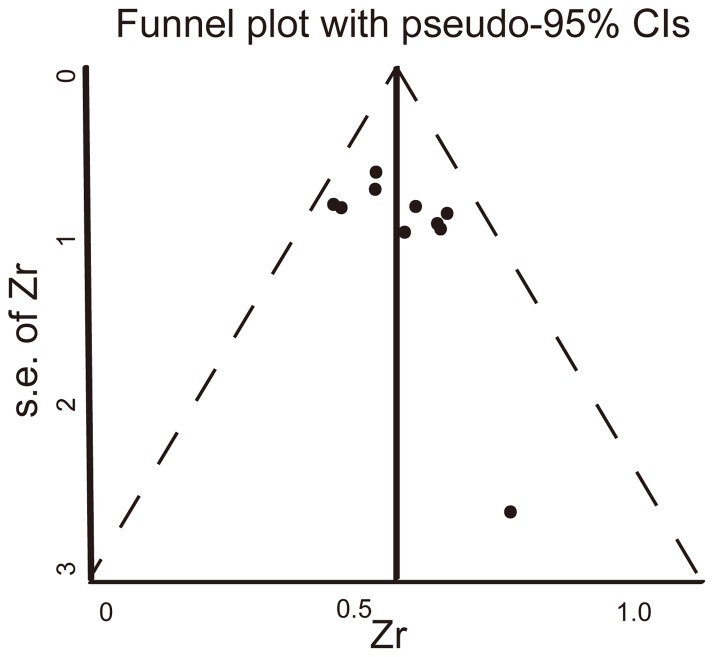
No publication bias was detected using a filled funnel plot with pseudo-95% (CIs) of results of 10 studies. Zr, transformed correlation coefficient (r); s.e., standard error.

For excluding heterogeneity, we testedonly the ten studies that detected LC in FS in the morning.The funnel plot and Egger's linear regression testdetected no publication bias among these studies (*P* = 0.053) (Figure 3).

## Discussion

The BMI allows health professionals to discuss overweight and underweight problems objectively with their patients [56]. The BMI has limitations but is used widely [57].

Most of the studies included in this meta-analysis involved blood collection in the early-to-late morning (06∶00 h to 9∶00 h). However, 4 studies did not specify the state (e.g., FS) at collection [22], [28], [30], [32] and 8 studies did not specify the time of blood collection [23], [28], [32], [35], [36], [39], [40], [46] and both factors were not specified in 3 studies [28], [32], [35]. These variations in the timing or status of specimen collection could significantly affect the obtained LC value in the studies.Licinio *et al*. found that serum LC exhibited a pattern of pulsatile release, with 32.0±1.5 pulses every 24 h and a pulse duration of 32.8±1.6 min in healthy men [58]. Adult men and women shared with a similar pulse frequency of leptin within 24 h even though the concentration was higher in women than in men [59]. Hence, the LC is dynamic, and timing can appreciably affect the measurement of leptin in the blood [10]. Leptin has a short half-time (about 5–7.5 min) [60], [61], Price *et al*. modified the leptin structure and expanded its half-time to 32.3 min [61]. Reports have clearly shown the leptin level to be lowest in the morning after an overnight fast [59], [62]–[64] and that LC increased after feeding [64], [65]. Our meta-analysis focused on the pre-analytical sources (BMI, age, HRT status) and analytical sources (analytical method, sampling time) affecting PW. The heterogeneity diminished if the analysis was conducted in a FS in the morning, suggesting that the LC is dynamic during the day. However, Hancox *et al*. reported no difference in the leptin concentration between semi-fasting and overnight fasting [66]. It could be concluded that plasma levels of leptin reflect primarily the total adipose mass rather than short FS, meal consumption or the dietary energy source [64], [66]. All of these sources of variation make it difficult to accurately determine a reference range of LC in healthy PW.

Interestingly, our analyses from all 15 studies that investigated both leptin concentration and BMI, showed clearly a positive relationship between LC and BMI (*P* = 0.025). At the same time we also should note that if sampling collection was restricted to the morning, the relationship between fasting LC and BMI just showed a trend (*P* = 0.299). We consider that for this no-significant relationship of FS LC and BMI, at least a possible reason is due to the reduced sample size (*n* = 10) for FS in the morning. Hence our results showed a moderate correlation between LC and BMI, serum LC increased significantly with the increase in BMI.

Our results showed that: (i) even though studies determined LC, the results were diverse and identifying a generic LC for PW according to criteria set by the Clinical and Laboratory Standards Institute is difficult; (ii) ELISA and RIA are used for the measurement of LC, but RIA is a more popular methodology that is recommended used to ascertain LC; (iii) LC has a wide concentration range in PW; (iv) obese PW had a higher mean value of LC; (v) undergoing HRT did not affect LC in PW; (vi) age affected LC only mildly. LC is higher in obese individuals than in non-obese subjects, andcan be found in other populations [1], [4].

A range of 12–14 h fasting time was stated in most of the included studies. FS and food intake are two other important pre-analytical variables that should be acknowledged. During FS, glycogen stores become the primary energy source for the body through glycogenolysis [10]. Fasting longer than 12–15 h results in the depletion of glycogen stores and a consequent increase in luconeogenesis [10]. However, different LC values in the FS could not be ascertained.

Leptin administration for the obese populationhas not shown encouraging results because of its short half-life in the circulation, low potency, and poor solubility [60].It was found that the metabolic effects of leptin act predominantly *via* the brain after leptin crosses the BBB by a saturable pattern, and that even peripheral leptin receptors exist [4], [67]. Leptin interacts with hypothalamic-pituitary-growth hormone as well as the hypothalamic–pituitary–adrenalandhypothalamic–pituitary–thyroid axes, and is involvedin glucose metabolism, reproduction, pubertal development, hematopoiesis and the immune system [4], [68], [69]. Leptin also has peripheral effects on skeletal muscle, the liver, pancreas and several other tissues [4], [68]. Systemic injection of leptin in mice or rats subjected to hyperinsulinemic clamp studies improved the effects of insulin and further decreased hepatic glucose production. After leptin was administered *via* the intracerebroventricular (ICV) route into the third ventricle at much lower doses in lean male rats, its metabolic effects could be almost replicated, also suggesting that leptin action in the brain is largely responsible for these effects. Furthermore, Fliedner *et al*. found that radiolabeled leptin preferentially reaches the hypothalamus and that hyperleptinemia could not block leptin transport to the brain. These findings suggested that the intranasal (IN) route of leptin administration could be a potential therapeutic method for obesity [70], but further works are needed to evaluate its effects. Clinical application of leptin was revised intensively by Yildiz *et al*. [67]and Scheler *et al*. [4].

The levels of some hormones keep changing during the postmenopausal process, especially in the early period (e.g., estrogen) [71], [72]. The morbidity due to hypertension [73], [74], cancer [75] and Alzheimer's disease [76] will increase with age. Recently, some reports showed that these diseases could be prevented after HRT [77], [78]. However, Marjoribanks *et al*. reported that HRT could prevent only postmenopausal osteoporosis [79], [80]. HRT usually involves three methods: estrogen, EPT, and progestin. In this meta-analysis, all of the PW who underwent treatment with EPT had LC values that did not change after HRT.

We were very cautious when comparing absolute LC values across studies by Nagy and Gower almost 10 year ago [81]. This conclusion was confirmed by two recent meta-analyses [49], [82] and our meta-analysis. Many sources of analytical variation will affect the accuracy of the standardization of LC measurement. Additionally, we analyzed the total LC in PW, but leptin mediates its effects after it binds to its receptors in tissues, so free leptin has not elicited its effects [83], [84]. An internationally accepted reference LC does not exist currently because of detection from different populations and different methods of analyses.

In conclusion, the present meta-analysis provides solid evidence in different population groups that LC values in PW are positively associated with the BMI and not associated with HRT.

## Supporting Information

Checklist S1 **Checklist of our manuscript.**
(DOC)

## References

[1] Cheung WW, Mao P (2012) Recent advances in obesity: genetics and beyond. ISRN Endocrinol 2012: 536905. 22474595 10.5402/2012/536905 PMC3313574

[2] Stunkard AJ, Foch TT, Hrubec Z (1986) A twin study of human obesity. JAMA 256: 51–54. 3712713

[3] Hjelmborg J, Fagnani C, Silventoinen K, McGue M, Korkeila M, et al (2008) Genetic influences on growth traits of BMI: a longitudinal study of adult twins. Obesity (Silver Spring) 16: 847–852. 18239571 10.1038/oby.2007.135

[4] Scherer T, Lehnert H, Hallschmid M (2013) Brain insulin and leptin signaling in metabolic control: from animal research to clinical application. Endocrinol Metab Clin North Am 42: 109–125. 23391243 10.1016/j.ecl.2012.11.002

[5] Zhang Y, Proenca R, Maffei M, Barone M, Leopold L, et al (1994) Positional cloning of the mouse obese gene and its human homologue. Nature 372: 425–432. 7984236 10.1038/372425a0

[6] Pralong FP, Gonzales C, Voirol MJ, Palmiter RD, Brunner HR, et al (2002) The neuropeptide Y Y1 receptor regulates leptin-mediated control of energy homeostasis and reproductive functions. FASEB J 16: 712–714. 11978737 10.1096/fj.01-0754fje

[7] Carlton ED, Demas GE, French SS (2012) Leptin, a neuroendocrine mediator of immune responses, inflammation, and sickness behaviors. Horm Behav 62: 272–279. 22561456 10.1016/j.yhbeh.2012.04.010

[8] Procaccini C, Lourenco EV, Matarese G, La Cava A (2009) Leptin signaling: A key pathway in immune responses. Curr Signal Transduct Ther 4: 22–30. 19774101 10.2174/157436209787048711 PMC2747760

[9] Oswal A, Yeo G (2010) Leptin and the control of body weight: a review of its diverse central targets, signaling mechanisms, and role in the pathogenesis of obesity. Obesity (Silver Spring) 18: 221–229. 19644451 10.1038/oby.2009.228

[10] Leibel RL (2002) The role of leptin in the control of body weight. Nutr Rev 60: S15-19; discussion S68–84, 85–17. 10.1301/002966402320634788 12403079

[11] Shanley LJ, Irving AJ, Harvey J (2001) Leptin enhances NMDA receptor function and modulates hippocampal synaptic plasticity. J Neurosci 21: RC186. 11734601 10.1523/JNEUROSCI.21-24-j0001.2001 PMC6763052

[12] Ray A (2012) Adipokine leptin in obesity-related pathology of breast cancer. J Biosci 37: 289–294. 22581334 10.1007/s12038-012-9191-9

[13] Sayakhot P, Vincent A, Teede H (2012) Breast cancer and menopause: perceptions of diagnosis, menopausal therapies and health behaviors. Climacteric 15: 59–67. 22132862 10.3109/13697137.2011.603772

[14] Thompson KJ, Lau KN, Johnson S, Martinie JB, Iannitti DA, et al (2011) Leptin inhibits hepatocellular carcinoma proliferation via p38-MAPK-dependent signalling. HPB (Oxford) 13: 225–233. 21418127 10.1111/j.1477-2574.2010.00259.x PMC3081622

[15] Wang SN, Lee KT, Ker CG (2010) Leptin in hepatocellular carcinoma. World J Gastroenterol 16: 5801–5809. 21155000 10.3748/wjg.v16.i46.5801 PMC3001970

[16] Saxena NK, Sharma D, Ding X, Lin S, Marra F, et al (2007) Concomitant activation of the JAK/STAT, PI3K/AKT, and ERK signaling is involved in leptin-mediated promotion of invasion and migration of hepatocellular carcinoma cells. Cancer Res 67: 2497–2507. 17363567 10.1158/0008-5472.CAN-06-3075 PMC2925446

[17] Ptak A, Kolaczkowska E, Gregoraszczuk EL (2012) Leptin stimulation of cell cycle and inhibition of apoptosis gene and protein expression in OVCAR-3 ovarian cancer cells. Endocrine43: 394–403. 10.1007/s12020-012-9788-7 PMC3593082 22968658

[18] Uddin S, Bu R, Ahmed M, Abubaker J, Al-Dayel F, et al (2009) Overexpression of leptin receptor predicts an unfavorable outcome in Middle Eastern ovarian cancer. Mol Cancer 8: 74. 19765303 10.1186/1476-4598-8-74 PMC2754986

[19] Siegel R, Naishadham D, Jemal A (2012) Cancer statistics, 2012. CA Cancer J Clin 62: 10–29. 22237781 10.3322/caac.20138

[20] Hedges LV, Olkin I, Statistiker M (1985) Statistical methods for meta-analysis. Academic Press New York.

[21] Hedges LV, Vevea JL (1998) Fixed-and random-effects models in meta-analysis. Psychol Methods 3: 486.

[22] Larsson H, Elmstahl S, Ahren B (1996) Plasma leptin levels correlate to islet function independently of body fat in postmenopausal women. Diabetes 45: 1580–1584. 8866564 10.2337/diab.45.11.1580

[23] Haffner SM, Mykkanen L, Stern MP (1997) Leptin concentrations in women in the San Antonio Heart Study: Effect of menopausal status and postmenopausal hormone replacement therapy. Am J Epidemiol 146: 581–585. 9326436 10.1093/oxfordjournals.aje.a009317

[24] Goulding A, Taylor RW (1998) Plasma leptin values in relation to bone mass and density and to dynamic biochemical markers of bone resorption and formation in postmenopausal women. Calcif Tissue Int 63: 456–458. 9817937 10.1007/s002239900557

[25] Hadji P, Gorke K, Hars O, Bauer T, Emons G, et al (2000) The influence of hormone replacement therapy (HRT) on serum leptin concentration in postmenopausal women. Maturitas 37: 105–111. 11137329 10.1016/s0378-5122(00)00166-3

[26] Hadji P, Hars O, Bock K, Sturm G, Bauer T, et al (2000) The influence of menopause and body mass index on serum leptin concentrations. Eur J Endocrinol 143: 55–60. 10870031 10.1530/eje.0.1430055

[27] Blain H, Vuillemin A, Guillemin F, Durant R, Hanesse B, et al (2002) Serum leptin level is a predictor of bone mineral density in postmenopausal women. J Clin Endocrinol Metab 87: 1030–1035. 11889157 10.1210/jcem.87.3.8313

[28] Douchi T, Iwamoto I, Yoshimitsu N, Kosha S, Nagata Y (2002) Leptin production in pre- and postmenopausal women. Maturitas 42: 219–223. 12161046 10.1016/s0378-5122(02)00078-6

[29] Hadji P, Bock K, Gotschalk M, Hars O, Backhus J, et al (2003) The influence of serum leptin concentration on bone mass assessed by quantitative ultrasonometry in pre and postmenopausal women. Maturitas 44: 141–148. 12590010 10.1016/s0378-5122(02)00324-9

[30] Lambrinoudaki I, Christodoulakos G, Panoulis C, Botsis D, Rizos D, et al (2003) Determinants of serum leptin levels in healthy postmenopausal women. J Endocrinol Invest 26: 1225–1230. 15055477 10.1007/BF03349162

[31] Munoz J, Gower BA (2003) Relationship between serum leptin concentration and low-density muscle in postmenopausal women. J Clin Endocrinol Metab 88: 1157–1161. 12629099 10.1210/jc.2002-020959

[32] Sahin G, Polat G, Baethis S, Milcan A, Baethdatoethlu O, et al (2003) Body composition, bone mineral density, and circulating leptin levels in postmenopausal Turkish women. Rheumatol Int 23: 87–91. 12634942 10.1007/s00296-002-0257-0

[33] Shaarawy M, Abassi AF, Hassan H, Salem ME (2003) Relationship between serum leptin concentrations and bone mineral density as well as biochemical markers of bone turnover in women with postmenopausal osteoporosis. Fertil Steril 79: 919–924. 12749431 10.1016/s0015-0282(02)04915-4

[34] Ushiroyama T, Ikeda A, Hosotani T, Higashiyama T, Ueki M (2003) Inverse correlation between serum leptin concentration and vertebral bone density in postmenopausal women. Gynecol Endocrinol 17: 31–36. 12724016

[35] Cordero-MacIntyre ZR, Metghalchi S, Rosen J, Peters W, Lohman TG, et al (2004) Impact of weight loss on serum leptin in obese postmenopausal women. J Appl Res 4: 60–67.

[36] Ayub N, Khan SR, Syed F (2006) Leptin levels in pre and post menopausal Pakistani women. J Pak Med Assoc 56: 3–5. 16454125

[37] Jaleel F, Jaleel A, Rahman MA, Alam E (2006) Comparison of adiponectin, leptin and blood lipid levels in normal and obese postmenopausal women. J Pak Med Assoc 56: 391–394. 17091749

[38] Rolland YM, Perry HM III, Patrick P, Banks WA, Morley JE (2006) Leptin and adiponectin levels in middle-aged postmenopausal women: associations with lifestyle habits, hormones, and inflammatory markers-a cross-sectional study. Metabolism 55: 1630–1636. 17142136 10.1016/j.metabol.2006.07.026

[39] Meyers JA, Liu AY, McTiernan A, Wener MH, Wood B, et al (2008) Serum leptin concentrations and markers of immune function in overweight or obese postmenopausal women. J Endocrinol 199: 51–60. 18614715 10.1677/JOE-07-0569

[40] Khokhar KK, Sidhu S, Kaur G (2010) Correlation between leptin level and hypertension in normal and obese pre- and postmenopausal women. Eur J Endocrinol 163: 873–878. 20841449 10.1530/EJE-10-0714

[41] Wu X-Y, Wu X-P, Luo X-H, Xie H, Zhang H, et al (2010) The relationship between the levels of gonadotropic hormones and OPG, leptin, TGF-beta 1 and TGF-beta 2 in Chinese adult women. Clinica Chimica Acta 411: 1296–1305. 10.1016/j.cca.2010.05.006 20478283

[42] Kristensen K, Pedersen SB, Vestergaard P, Mosekilde L, Richelsen B (1999) Hormone replacement therapy affects body composition and leptin differently in obese and non-obese postmenopausal women. J Endocrinol 163: 55–62. 10495407 10.1677/joe.0.1630055

[43] Cagnacci A, Malmusi S, Arangino S, Zanni A, Rovati L, et al (2002) Influence of transdermal estradiol in the regulation of leptin levels of postmenopausal women: a double-blind, placebo-controlled study. Menopause (New York, NY) 9: 65–71. 10.1097/00042192-200201000-00010 11791088

[44] Carlo C, Tommaselli GA, Sammartino A, Bifulco G, Nasti A, et al (2004) Serum leptin levels and body composition in postmenopausal women: effects of hormone therapy. Menopause (New York, NY) 11: 466–473. 10.1097/01.gme.0000109313.11228.2b 15243285

[45] Carlo C, Tommaselli GA, Spiezio Sardo A, Sammartino A, Attianese W, et al (2007) Longitudinal evaluation of serum leptin and bone mineral density in early postmenopausal women. Menopause (New York, NY) 11: 450–454. 10.1097/01.gme.0000236936.28454.6a 17242633

[46] Soni AC, Conroy MB, MacKey RH, Kuller LH (2011) Ghrelin, leptin, adiponectin, and insulin levels and concurrent and future weight change in overweight, postmenopausal women. Menopause 18: 296–301. 21449093 10.1097/gme.0b013e3181f2e611 PMC3069721

[47] Di Carlo C, Tommaselli GA, Pisano G, Nasti A, Rossi V, et al (2000) Serum leptin levels in postmenopausal women: effects of transdermal hormone replacement therapy. Menopause (New York, NY) 7: 36–41. 10.1097/00042192-200007010-00007 10646702

[48] Bednarek-Tupikowska G, Filus A, Kuliczkowska-Plaksej J, Tupikowski K, Bohdanowicz-Pawlak A, et al (2006) Serum leptin concentrations in pre- and postmenopausal women on sex hormone therapy. Gynecol Endocrinol 22: 207–212. 16723307 10.1080/09513590600702774

[49] Ren RX, Shen Y (2010) A meta-analysis of relationship between birth weight and cord blood leptin levels in newborns. World J Pediatr 6: 311–316. 20549405 10.1007/s12519-010-0216-x

[50] Rozenberg S, Vandromme J, Antoine C (2013) Postmenopausal hormone therapy: risks and benefits. Nat Rev Endocrinol 9: 216–227. 23419265 10.1038/nrendo.2013.17

[51] Simon JA (2010) Postmenopausal hormone therapy: risks versus benefits reassessed. Nat Rev Endocrinol 6: 661–663. 21102647 10.1038/nrendo.2010.191

[52] Prentice RL, Manson JE, Langer RD, Anderson GL, Pettinger M, et al (2009) Benefits and risks of postmenopausal hormone therapy when it is initiated soon after menopause. Am J Epidemiol 170: 12–23. 19468079 10.1093/aje/kwp115 PMC2733042

[53] Banks E, Canfell K (2009) Invited Commentary: Hormone therapy risks and benefits–The Women's Health Initiative findings and the postmenopausal estrogen timing hypothesis. Am J Epidemiol 170: 24–28. 19468078 10.1093/aje/kwp113

[54] van Staa TP, Cooper C, Barlow D, Leufkens HG (2008) Individualizing the risks and benefits of postmenopausal hormone therapy. Menopause 15: 374–381. 17882009 10.1097/gme.0b013e31812e558f

[55] Manson JE (2013) The role of personalized medicine in identifying appropriate candidates for menopausal estrogen therapy. Metabolism 62 Suppl 1S15–19. 23018143 10.1016/j.metabol.2012.08.015

[56] MacKay NJ (2010) Scaling of human body mass with height: the body mass index revisited. J Biomech 43: 764–766. 19909957 10.1016/j.jbiomech.2009.10.038

[57] Flegal KM, Troiano RP, Ballard-Barbash R (2001) Aim for a healthy weight: what is the target? J Nutr 131: 440S–450S. 11160576 10.1093/jn/131.2.440S

[58] Licinio J, Mantzoros C, Negrao AB, Cizza G, Wong ML, et al (1997) Human leptin levels are pulsatile and inversely related to pituitary-adrenal function. Nat Med 3: 575–579. 9142131 10.1038/nm0597-575

[59] Licinio J, Negrao AB, Mantzoros C, Kaklamani V, Wong ML, et al (1998) Sex differences in circulating human leptin pulse amplitude: clinical implications. J Clin Endocrinol Metab 83: 4140–4147. 9814504 10.1210/jcem.83.11.5291

[60] Vila R, Adan C, Rafecas I, Fernandez-Lopez JA, Remesar X, et al (1998) Plasma leptin turnover rates in lean and obese Zucker rats. Endocrinology 139: 4466–4469. 9794453 10.1210/endo.139.11.6296

[61] Price TO, Farr SA, Yi X, Vinogradov S, Batrakova E, et al (2010) Transport across the blood-brain barrier of pluronic leptin. J Pharmacol Exp Ther 333: 253–263. 20053933 10.1124/jpet.109.158147 PMC2846026

[62] Langendonk JG, Pijl H, Toornvliet AC, Burggraaf J, Frolich M, et al (1998) Circadian rhythm of plasma leptin levels in upper and lower body obese women: influence of body fat distribution and weight loss. J Clin Endocrinol Metab 83: 1706–1712. 9589680 10.1210/jcem.83.5.4717

[63] Koutkia P, Canavan B, Johnson ML, DePaoli A, Grinspoon S (2003) Characterization of leptin pulse dynamics and relationship to fat mass, growth hormone, cortisol, and insulin. Am J Physiol Endocrinol Metab 285: E372–379. 12721156 10.1152/ajpendo.00097.2003

[64] Weigle DS, Duell PB, Connor WE, Steiner RA, Soules MR, et al (1997) Effect of fasting, refeeding, and dietary fat restriction on plasma leptin levels. J Clin Endocrinol Metab 82: 561–565. 9024254 10.1210/jcem.82.2.3757

[65] Bergendahl M, Iranmanesh A, Evans WS, Veldhuis JD (2000) Short-term fasting selectively suppresses leptin pulse mass and 24-hour rhythmic leptin release in healthy midluteal phase women without disturbing leptin pulse frequency or its entropy control (pattern orderliness). J Clin Endocrinol Metab 85: 207–213. 10634388 10.1210/jcem.85.1.6325

[66] Hancox RJ, Landhuis CE (2011) Correlation between measures of insulin resistance in fasting and non-fasting blood. Diabetol Metab Syndr 3: 23. 21899745 10.1186/1758-5996-3-23 PMC3177770

[67] Yildiz BO, Haznedaroglu IC (2006) Rethinking leptin and insulin action: therapeutic opportunities for diabetes. Int J Biochem Cell Biol 38: 820–830. 16236542 10.1016/j.biocel.2005.09.013

[68] Friedman JM (2002) The function of leptin in nutrition, weight, and physiology. Nutr Rev 60: S1-14; discussion S68–84, 85–17. 10.1301/002966402320634878 12403078

[69] Himms-Hagen J (1999) Physiological roles of the leptin endocrine system: differences between mice and humans. Crit Rev Clin Lab Sci 36: 575–655. 10656540 10.1080/10408369991239259

[70] Fliedner S, Schulz C, Lehnert H (2006) Brain uptake of intranasally applied radioiodinated leptin in Wistar rats. Endocrinology 147: 2088–2094. 16469809 10.1210/en.2005-1016

[71] Chahal HS, Drake WM (2007) The endocrine system and ageing. J Pathol 211: 173–180. 17200939 10.1002/path.2110

[72] Taylor GT, Bardgett M, Farr S, Womack S, Komitowski D, et al (1993) Steroidal interactions in the ageing endocrine system: absence of suppression and pathology in reproductive systems of old males from a mixed-sex socially stressful rat colony. J Endocrinol 137: 115–122. 8492069 10.1677/joe.0.1370115

[73] Coylewright M, Reckelhoff JF, Ouyang P (2008) Menopause and hypertension: an age-old debate. Hypertension 51: 952–959. 18259027 10.1161/HYPERTENSIONAHA.107.105742

[74] Rappelli A (2002) Hypertension and obesity after the menopause. J Hypertens Suppl 20: S26–28. 12183846

[75] Britt K (2012) Menarche, menopause, and breast cancer risk. Lancet Oncol 13: 1071–1072. 23084520 10.1016/S1470-2045(12)70456-4

[76] Casadesus G, Rolston RK, Webber KM, Atwood CS, Bowen RL, et al (2008) Menopause, estrogen, and gonadotropins in Alzheimer's disease. Adv Clin Chem 45: 139–153. 18429496 10.1016/s0065-2423(07)00006-6

[77] Davey DA (2012) Update: estrogen and estrogen plus progestin therapy in the care of women at and after the menopause. Womens Health (Lond Engl) 8: 169–189. 22375720 10.2217/whe.12.1

[78] Hinds L, Price J (2010) Menopause, hormone replacement and gynaecological cancers. Menopause Int 16: 89–93. 20729501 10.1258/mi.2010.010018

[79] Marjoribanks J, Farquhar C, Roberts H, Lethaby A (2012) Trial does not change the conclusions of Cochrane review of long term hormone therapy for perimenopausal and postmenopausal women. BMJ 345: e8141. 23208255 10.1136/bmj.e8141

[80] Marjoribanks J, Farquhar C, Roberts H, Lethaby A (2012) Long term hormone therapy for perimenopausal and postmenopausal women. Cochrane Database Syst Rev 7: CD004143. 10.1002/14651858.CD004143.pub4 22786488

[81] Nagy TR, Gower BA, Trowbridge CA, Dezenberg C, Shewchuk RM, et al (1997) Effects of gender, ethnicity, body composition, and fat distribution on serum leptin concentrations in children. J Clin Endocrinol Metab 82: 2148–2152. 9215286 10.1210/jcem.82.7.4077

[82] Venner AA, Doyle-Baker PK, Lyon ME, Fung TS (2009) A meta-analysis of leptin reference ranges in the healthy paediatric prepubertal population. Ann Clin Biochem 46: 65–72. 19103960 10.1258/acb.2008.008168

[83] Kelesidis T, Kelesidis I, Chou S, Mantzoros CS (2010) Narrative review: the role of leptin in human physiology: emerging clinical applications. Ann Intern Med 152: 93–100. 20083828 10.1059/0003-4819-152-2-201001190-00008 PMC2829242

[84] Brennan AM, Mantzoros CS (2006) Drug Insight: the role of leptin in human physiology and pathophysiology–emerging clinical applications. Nat Clin Pract Endocrinol Metab 2: 318–327. 16932309 10.1038/ncpendmet0196

